# A Refined Neuronal Population Measure of Visual Attention

**DOI:** 10.1371/journal.pone.0136570

**Published:** 2015-08-21

**Authors:** J. Patrick Mayo, Marlene R. Cohen, John H. R. Maunsell

**Affiliations:** 1 Department of Neurobiology, Harvard Medical School, 220 Longwood Avenue, Boston, Massachusetts, United States of America; 2 Department of Neuroscience and Center for the Neural Basis of Cognition, University of Pittsburgh, Pittsburgh, Pennsylvania, United States of America; 3 Department of Neurobiology, University of Chicago, 5812 S. Ellis Avenue, Chicago, Illinois, United States of America; University of Verona, ITALY

## Abstract

Neurophysiological studies of cognitive mechanisms such as visual attention typically ignore trial-by-trial variability and instead report mean differences averaged across many trials. Advances in electrophysiology allow for the simultaneous recording of small populations of neurons, which may obviate the need for averaging activity over trials. We recently introduced a method called the attention axis that uses multi-electrode recordings to provide estimates of attentional state of behaving monkeys on individual trials. Here, we refine this method to eliminate problems that can cause bias in estimates of attentional state in certain scenarios. We demonstrate the sources of these problems using simulations and propose an amendment to the previous formulation that provides superior performance in trial-by-trial assessments of attentional state.

## Introduction

The advent of multi-channel microelectrode arrays has made it easier to simultaneously record from small populations of neurons. The richness of the data obtained from arrays requires appropriate analyses for describing a population of neurons that encodes sensory, motor, or cognitive information (the “population code”). Various techniques have been used to characterize a neuronal population’s responses [1–4].

We recently introduced a method for using population activity to estimate the attentional state of subjects: the “attention axis” [5]. We recorded extracellularly from small populations of neurons in visual area V4 during a change-detection task that required spatial attention. The attention axis allowed us to infer the attentional state of the animal within relatively short periods, including single trials and individual stimulus presentations of only 200 ms. We found that on trials in which the neurons indicated that the monkey was attending strongly to one location, the monkey was better able to detect stimulus changes at that location. Thus, the attention axis might advance our ability to study visual attention and other cognitive processes at behaviorally relevant timescales.

However, we have since discovered that certain biologically plausible scenarios can lead to distortions in the attention axis analysis that can bias measurements of cognitive state. Two different factors, in particular, can lead to apparent changes in position on the attention axis where none exist. First, when the attention axis is constructed using populations of neurons with responses that differ little between different attentional conditions, measurements can be biased toward the unattended location. Second, a bias in the same direction can occur when the attention axis is constructed using small samples of neuronal responses that provide poor estimates of the underlying activity. It is important to identify and address the sources of these distortions to ensure relatively unbiased estimates of neuronal activity and behavior.

We describe here a refinement of the attention axis that effectively eliminates these biases by equating them across behavioral conditions and thereby minimizing their contributions to our results. For comparison, we re-compute previously reported results using our improved metric. The corrected measures show somewhat less variance in attentional state overall, but the refined attention axis retains its ability to capture moment-by-moment fluctuations in attention that otherwise would be undetected by conventional trial-averaging measures.

## Materials and Methods

Because the construction of the attention axis is the focus of this work, we briefly outline the analysis below. Details were provided in previous work [1].

**Fig 1:**
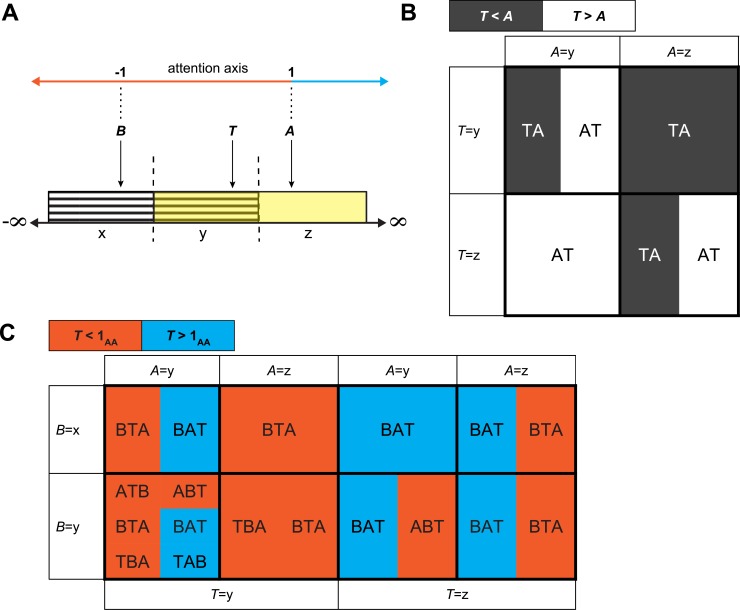
Attention axis construction and outcomes. **(A)** Schematic of two overlapping uniform distributions (hatched and yellow). Samples *A* and *T* are always drawn from the yellow distribution and can be located in range y or z. Sample *B* is drawn from the hatched distribution and can be located in range x or y. Samples A and B are used to construct an attention axis, which is normalized to *A* = 1 and *B* = -1. **(B)** Representation of possible outcomes and probabilities given samples *A* and *T* only. Fill colors show probabilities of sample *T* being less (black) or greater (white) than *A*. Unbiased sampling leads to equal total areas for black and white cells. **(C)** Representation of all possible outcomes for the value of *T* on the attention axis. Same conventions as B, except probabilities are relative to 1 on the attention axis. The excess of orange relative to blue indicates that sampling is biased towards values of *T* that are less than 1 on the attention axis.

Monkeys were trained to attend to one of two stimuli, flashed on and off simultaneously, and to respond with an eye movement to a stimulus when one stimulus changed its orientation. During each daily session, we recorded simultaneously from approximately 40 single units and multiunit clusters in each hemisphere of visual area V4 using chronically implanted multi-electrode arrays. One stimulus was located in each visual hemifield, in the receptive fields of neurons recorded in the contralateral hemisphere. To build the attention axis, neuronal responses to the stimulus presentations immediately preceding the stimulus change (stimulus “n-1”) were sampled on individual trials and plotted in N-dimensional space, where N is the number of simultaneously recorded single units and multiunit clusters. Trials in which the monkey correctly detected the stimulus change (“Hits”) at the cued location were divided into two conditions, “attend-left” and “attend-right”, based on where the animal was cued to attend to at the start of each block of trials. The line connecting the means of neuronal responses on Hit trials in each attention condition was defined as the original attention axis [5]. Neuronal responses from correctly detected changes (“Hits”) and missed changes (“Misses”) were then projected onto the attention axis and individual projections (i.e., the position on the attention axis) were used to infer the animals’ trial-by-trial state of attention. Mean activity in the attend-left and attend-right conditions was normalized to -1 and 1, respectively, to facilitate comparisons across recording sessions (e.g., Fig 1A, top). The revised attention axis is broadly applicable to simultaneous neuronal recordings in any brain region and from any microelectrode technology. It does not require simultaneous recordings from both brain hemispheres [5]. Also, the attention axis treats every neuron in the same manner regardless of cell type, and it does not take into account noise correlations or other forms of neuronal interactions.

Two adult rhesus macaques, *Macaca mulatta*, weighing 9 and 12 kg, were purchased from the New England Primate Research Center and pair-housed in Harvard Medical School’s animal facilities in accordance with University policies and the USA Public Health Service Guide for the Care and Use of Laboratory Animals. Neurophysiological data re-analyzed in this paper are from experiments approved by and conducted under the auspices of the Institutional Animal Care and Use Committee of Harvard Medical School [5, 6]. Monkeys were fed nutrient-rich biscuits as well as an assortment of supplemental treats (e.g., bananas, raisins, peanuts) daily. Enrichment activities typically included foraging for treats, music, movies, human interaction, and standard toys including mirrors. Animal health was monitored daily by trained professionals. The effects of water restriction were monitored closely by checking the weight, stool, and behavior of the animals; significant deviations of any of these factors led to *ad libitum* water access until symptoms resolved. Animals worked to satiation in the laboratory or were supplemented with water in their cages. Loose restraints were used to guide the animals into the chairs. Time in the laboratory and exposure to the visual attention task were increased gradually over the course of several weeks using operant conditioning and only positive reinforcement. Daily experimental sessions were terminated when the animals lost interest in performing the task.

Simulations were carried out in Matlab 2012a and collection of physiological data was previously described [5].

## Results

**Fig 2:**
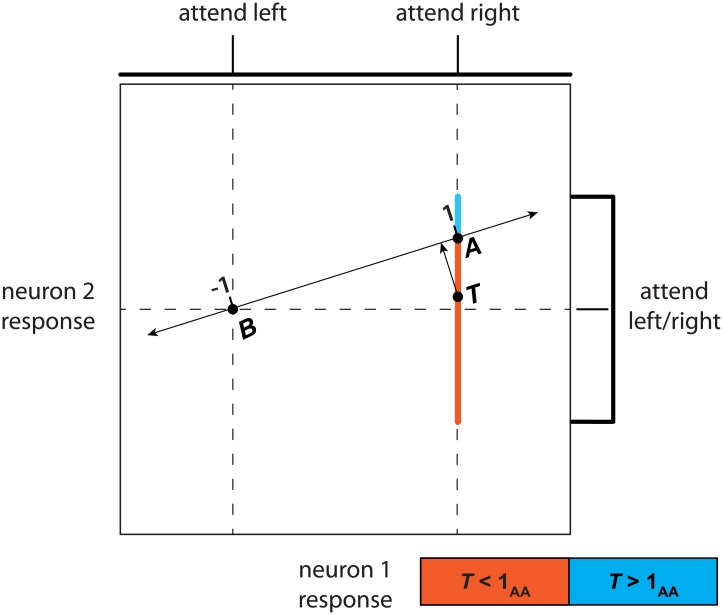
Multidimensional regression. The responses of two neurons in two conditions, attend-left and attend-right. Neuron 1’s response to attend-right is greater than that to attend-left, and its responses in both conditions are noiseless, as illustrated by the impulse functions (top) and vertical dashed lines. The response of neuron 2 is noiseless in the attend-left condition, but drawn from a uniform distribution in the attend-right condition (impulse function and spanned range on right). The attention axis (double-sided arrow) connects samples *A* and *B*.

**Fig 3:**
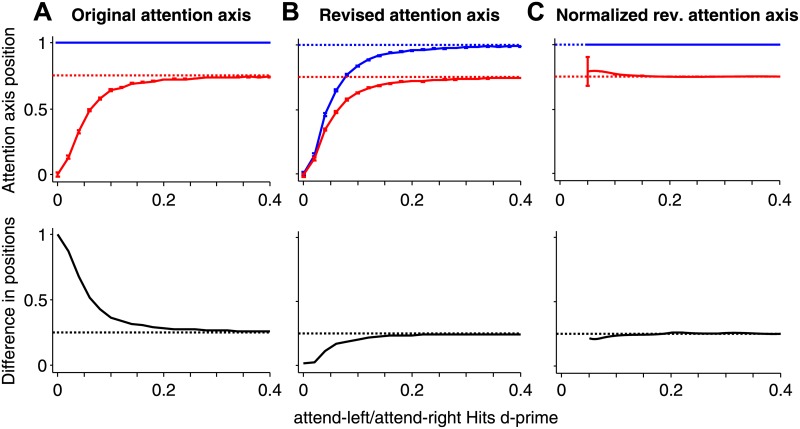
Average Hit (blue) and Miss (red) positions on the original (A), revised (B), and normalized revised (C) attention axes for attend-right responses only. Correct values are indicated by dotted lines. The original attention axis **(A)** overestimates differences (black, bottom) between Hits and Misses when modulation by attention is weak. The refined attention axis **(B)** underestimates the true separation of 0.25 (dotted line) when modulation by attention is weak. **(C)** Re-normalizing the revised attention axis makes it easier to estimate Hit-Miss differences at modulations greater than a d' of ~0.1. Error bars (top row) are standard error and usually less than the thickness of the line itself.

**Fig 4:**
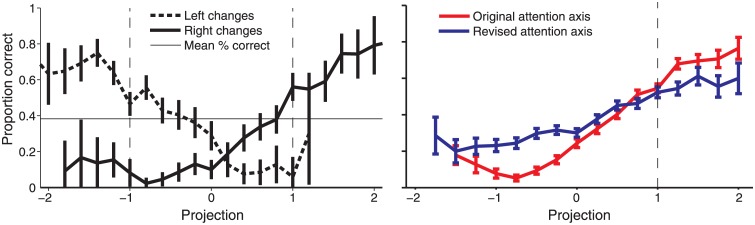
Behavioral performance as a function of attention axis position. *Left*, Projections on the original attention axis for each stimulus location (Fig 2F from [5]). Horizontal line indicates mean proportion correct across trials. *Right*, Same data combined across stimulus locations plotted on the original attention axis (red line) and re-plotted on the revised attention axis (blue line).

**Fig 5:**
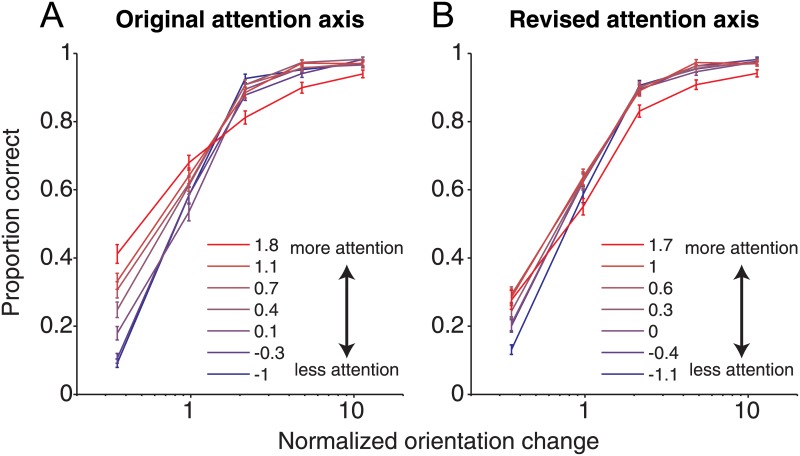
Psychometric curves as a function of attention axis position using the original (*A*; compare with Fig 1B from [6]) and revised (right) attention axes. Orientation changes were normalized to the behavioral threshold (63% correct) for each recording session and then binned into five equally sized bins. Trials were placed into seven equally sized bins according to position on the attention axis ranging from strong (red) to weak (blue) attentional modulation.

**Fig 6:**
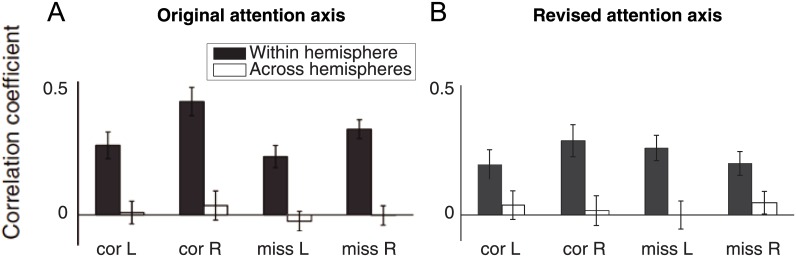
Attention axis positions are correlated when the axis is constructed using neurons recorded from the same hemisphere, but uncorrelated for those across left (L) and right (R) hemispheres. **(A)**, original attention axis, Fig 6 from [5]. **(B)**, revised attention axis.

The attention axis measures the attentional state of the subject during a brief period (here, 200 ms) based on the average modulation of neuronal responses between different attention conditions (e.g., attend-left vs. attend-right). Below, we illustrate potential problems with this measure as it was originally applied [5, 6]. The first two issues (Figs 1 and 2) can generate different projections on Hit and Miss trials even when there is no meaningful change in the underlying neuronal activity. These issues can be eliminated by using the modified analysis described below. We verify this refined approach using simulations (Fig 3) and discuss limitations of the sensitivity of attention axis measurements. Finally, we re-plot central results from our previous work using the refined attention axis (Figs 4–6).

The first potential problem with the attention axis occurs when the responses used to construct the axis are overlapping and the estimates of the response means are noisy. We demonstrate this problem in simulations using a highly reduced case in which the axis is constructed using a single sample from attend-right responses (*A*) and a single sample from attend-left responses (*B*) of a single neuron. In this example, the response estimates *A* and *B* are random samples drawn from two uniform distributions that overlap by 50% (Fig 1A, hatched and yellow). A single-trial test sample (*T*) is also drawn from the attend-right distribution. If the attention axis provides unbiased measures, the expected position for the test sample *T* on the attention axis must be 1, because it is drawn from the same distribution as *A*. We will show that this is not the case.

Samples *A*, *B*, and *T* might lie anywhere within their respective distributions. Here, for simplicity we distinguish cases where the samples lie in the left or right halves of the two distributions (labeled x, y and z in Fig 1A). By construction, *A* and *T* are equally likely to lie in y and z, and *B* is equally likely to lie in x or y. The probability that *T* < *A* is 0.5. Fig 1B illustrates specific outcomes for the locations of *A* and *T* and their probabilities. Because *A* and *T* are individually equally likely to fall in range y or z, the area of each of the four cells in Fig 1B corresponds to a probability of 0.25. When *T* is in y and *A* is in z, *T* will always lie to the left of *A* (black in Fig 1B). When *T* is in z and *A* is in y, *T* will always lie to the right of *A* (white in Fig 1B). When *A* and *T* are both in the same range (e.g., both in y), *T* < *A* and *T* > *A* are equally likely (the upper-left and lower-right cells in Fig 1B are half black and half white). Across all possible outcomes the black and white areas are equal, indicating that overall the probabilities *T* < *A* and *T* > *A* are each 0.5.

Although *T* is equally likely to lie to the left or right of *A*, it is not equally likely to lie in positions that are greater or less than 1 on the attention axis (Fig 1C). The symmetry of *T* relative to 1 on the attention axis holds when *B* is in x and is therefore always less than *A* (Fig 1C, top row). In this case, the possible outcomes and probabilities on the attention axis are identical to those in Fig 1B; there is equal probability of *T* taking a value greater than or less than 1 on the attention axis, as indicated by the equal blue and orange areas in the top row of Fig 1C.

The bias occurs when *A* and *B* both lie in range y, where *B* can be greater than *A*. Such inversions reverse the polarity of the attention axis, such that sample values toward the left map to more positive attention axis positions than those toward the right. Axis inversions occur whenever B lies to the right of A. Inversions account for the outcomes labeled ABT where the blue color in the upper row of Fig 1C (T > 1_AA_) is replaced by orange in the lower row (T < 1_AA_). When *A* and *B* are both in y and *T* is in z (more positive than both *A* and *B*), *T* will have a position on the attention axis that is greater than 1 when *A* < *B*, but equally often it will have a position on the attention axis that is less than 1, when *B* < *A* (third cell in bottom row of Fig 1C). Additionally, there are six equally probable arrangements when *A*, *B*, and *T* are all in range y (leftmost cell in bottom row of Fig 1C). But only two of these six arrangements result in situations where *T* > 1 on the attention axis. Under these conditions, the overall probability that T will have a value less than 1 on the attention axis in Fig 1C is 0.583 (0.5 plus the bias caused by outcomes ABT, 1/16 + 1/48). The ability of the orientation of the attention axis to flip in certain configurations means that there will be a bias for *T* to have a value shifted from 1 toward the direction of 0, even though *T* was drawn from the same distribution as *A*.

This bias in the attention axis stems from the overlap between distributions used to construct it and noisy estimates of the distribution means. If the estimates *A*, *B* and *T* were each based on a large number of samples, or individual responses from a large number of neurons, they would approximate the true means of the distributions, *B* would virtually never lie to the right of *A*, and the bias would be effectively eliminated. The amount of bias in the example in Fig 1 can be precisely calculated because the distributions have well-defined properties. To validate the probabilities in Fig 1C, we simulated the sampling scenario 10,000 times. The mean probability that *T* < 1 was 0.583 (SE 0.002) when *A* and *T* were randomly drawn from random uniform distributions (Fig 1C), while the mean probability that *T* < *A* was 0.50 (SE 0.002, Fig 1B), matching theoretical expectations. Thus, we can account for biased sampling on the attention axis when the statistics of the distributions are known, and potentially correct for it.

In practice, however, the attention axis is built on responses from many neurons that have distributions that can only be estimated. It is therefore difficult to know how much bias might enter into attention axis measurements. If the distributions of firing rates of the two response distributions that comprise the attention axis are non-overlapping, then axis inversion is avoided. But because neurons are typically weakly modulated by attention and are often driven by suboptimal sensory stimuli in neurophysiological experiments, overlapping population responses are common.

Although the bias described in Fig 1 does not occur if samples are drawn from two non-overlapping distributions, a second bias can occur even when the sample distributions do not overlap and is related to the process of projecting points in multidimensional space. This bias acts in the same direction as that described above, shifting measurements towards 0 on the attention axis. Fig 2 illustrates this other source of bias using a simplified example in which the population responses are based on responses from only two neurons. All responses are noiseless except the response of neuron 2 in the attend-right condition, which is drawn from a uniform distribution (Fig 2, "attend right").

As with the simulations in Fig 1, the attention axis is constructed using only a single sample of the neurons' responses from the attend-right response distribution, *A*, and a single sample of the neurons' responses from the attend-left response distribution, *B* (which is noiseless in this example). A single test sample, *T*, is drawn from the attend-right distribution and projected onto the attention axis. When neuron 2's response to *A* is greater than its mean value and its response to *T* is less than its response to *A*, then the projected position of *T* (black arrow) will be less than 1 on the attention axis (orange line segment). Alternatively, if *T* > *A* in that situation, then the projection is greater than 1 (blue vertical segment). As indicated by the greater length of the orange segment, test values are more likely to project to positions less than 1 on the attention axis. This bias favoring values less than 1 will occur whether the slope of the attention axis is positive or negative because the longer segment will project to attention axis values that are less than 1 in either case (the orange segment would be above the blue segment when the slope is negative). Thus, noisy estimates of the population means can artificially bias attention axis positions towards zero, even in non-overlapping distributions.

We simulated the scenario in Fig 2 with neuron 1’s responses fixed one unit apart (abscissa) and neuron 2’s attend-right response drawn from a uniform distribution one unit in extent (blue and orange vertical bars combined). The magnitude of the bias depended on the modulation by attention relative to the magnitude of the response noise. In the configuration illustrated in Fig 2, the mean position of sample *T* on the attention axis was 0.856 (SE 0.001), a bias towards 0. Doubling the extent of the uniform distribution increased the bias so that the mean position of *T* was 0.571 (SE 0.002). In contrast, doubling the difference between neuron 1’s responses (i.e., by moving *B* leftward in Fig 2) reduced the original bias such that the mean axis position was 0.96. The bias towards zero therefore increases as either 1) population responses become more variable, or 2) attentional modulation decreases.

The original attention axis was constructed using correct responses (“Hits”) from the epoch immediately before the stimulus change (“n-1”) in each trial. Because only Hits were used to construct the axis, the mean of Hit trials for the attend-right condition was forced to lie at 1, and that for the attend-left conditions was forced to lie at -1. Because the axis was constructed with a finite number of attend-left and attend-right Hit trial responses that had overlapping distributions [5], other responses projected onto the axis were susceptible to the biases described above (Figs 1 and 2). When the n-1 attend-left and attend-right Miss trials were projected onto the axis, these biases would shift their means toward 0 by some amount.

Without precise knowledge of the response distributions, it is impossible to eliminate these biases. However, the effect of the biases can be eliminated by ensuring that it acts equally on the responses being compared, for example, by constructing the attention axis using the responses to one particular stimulus on Hit trials, and then comparing the responses on Hit and Miss trials to a different stimulus. We did this by constructing the axis using responses to the stimulus that came two before the stimulus change on each trial (stimulus "n-2"). Hit and Miss responses to stimulus n-1 were then projected onto the axis to yield the attention axis position. Although n-1 responses projected onto the n-2 attention axis will have the biases described above, these biases will affect Hit and Miss trials equally, making it possible to accurately compare estimates of attention on trials with different behavioral outcomes.

The revised attention axis does not introduce artifactual differences between estimates for Hits and Misses because any bias in attention axis measurements acts on both equally. This fact is confirmed in the simulations shown in Fig 3. We fixed the mean of responses on Miss trials at 1/8^th^ of the distance between mean attend-left and mean attend-right Hit responses, and then varied attentional modulation. The expected difference between attend-right Hit and Miss projections was therefore always 0.25. We simulated the n-1 and n-2 responses of 20 neurons over 1000 trials. Responses were modeled as normal distributions and we varied the distance between mean attend-left and attend-right Hits (d' values: 0–0.4) to study the effect of attentional modulation on attention axis position. We measured the average difference between attend-right Hit and Miss positions on the original (Fig 3A) and revised (Fig 3B and 3C) attention axes after 1,000–1,000,000 repetitions at each d' value.

Under these conditions, the original attention axis performs well when the distributions of attend-left and attend-right responses of individual neurons differ by more than a d' of about 0.2. At smaller differences the mean Hit responses remain at 1 (by construction), but the biases described above cause the mean Miss responses to take values that are increasingly biased toward 0 as modulation by attention approaches 0.

For the revised attention axis (Fig 3B), the average attend-right Hit and Miss positions are asymptotically close to the correct positions of 1.00 and 0.75 when d' values are 0.2 and greater (Fig 3B, bottom). However at smaller d' values, Hit and Miss values both approach 0 as d' approaches 0 (Fig 3B, top). This inevitably leads to an underestimate of their difference, particularly when modulation by attention is small relative to the response noise. Thus, the revised attention axis leads to conservative measures of Hit-Miss differences when those differences in activity are very small (Fig 3B, bottom).

Because the bias makes absolute measures on the attention axis uninformative, it can be helpful to re-normalize the mean attend-right Hits to 1 (and attend-left Hits to -1) so that the bias underlying both Hits and Misses is effectively removed. This approach produces a more convenient measure of population responses for d-primes ≥ 0.05 (Fig 3C) that is still comparable to the original attention axis (Fig 3A), which tended to overestimate the true strength of modulation by attention. While helpful, this renormalization cannot address the unreliability of measures when attention-related modulation is small relative to uncertainty in estimates of individual neuronal population responses.

To illustrate the effectiveness of the refined attention axis using real data, we re-examined three key analyses that were previously presented using the original attention axis [5, 6]. Side-by-side comparisons of the original and revised attention axes are shown below (Figs 4–6).

If the attention axis is a useful measure of the animal’s attentional state, then position on the attention axis should correlate with behavioral performance. Alternatively, if axis position and task performance are largely unrelated, then the attention axis fails to capture neuronal activity that supports visual attention in our task. Fig 4 illustrates changes in behavioral performance as a function of projected position on the attention axis. Each trial was assigned to a bin by projecting onto the attention axis the neuronal population response to the stimulus immediately before the change appeared. The probability of successfully detecting the change was then computed for each bin. Animals performed well when population responses on the original attention axis (Fig 4, left) were at the mean hit response positions and beyond (less than -1 and greater than 1 for attend-left and attend-right conditions, respectively). Performance decreased as position on the attention axis approached and moved beyond zero. These results suggest that the position on the original attention axis can be used to predict the animal’s state of attention. But given the biases in the original attention axis, it is unclear how much of the result can be attributed to true changes in neuronal population responses. The bias would cause Miss trials, but not Hit trials, to be assigned to positions on the attention axis that are shifted toward 0, making the proportion correct (Hit trials / Hit trials + Miss trials) smaller for attention axis positions further from 1 or -1.

To compare the same data on the original and revised attention axes, we combined data across attend-right and attend-left conditions by normalizing the mean of the Hit distributions in each attention condition to 1 (Fig 4, right). The relationship between attention axis position and performance is weaker when using the revised attention axis compared to the original attention axis, as indicated by the decrease in slope. This suggests that bias contributed some fraction of the effects previously reported. However, a clear relationship exists on both axes: task performance was high at positions greater than 1 (which we hypothesize correspond to trials in which the animal correctly allocated attention to the stimulus where the orientation change occurred), and performance steadily decreased as positions approached zero and beyond. The systematic change in behavioral performance as a function of position on the revised attention axis demonstrates that the refined attention axis captures substantial task-relevant changes in attentional state.

Given that position on the attention axis was a reliable predictor of trial-by-trial behavioral performance [5], we also previously examined whether attention improved performance by decreasing the threshold or slope of psychometric functions [6]. The influence of changes in population activity on these two behavioral parameters might help us better understand the neuronal mechanisms that allow for improved performance associated with attention. Using the original attention axis, we found that both the threshold and the slope of psychometric functions decreased as the strength of attention increased (Fig 5A). We reanalyzed the data using the revised attention axis and found that these findings hold true (Fig 5B), although both changes were reduced.

Changes in population activity, as measured by the attention axis, can help elucidate not only how neuronal responses support behavior but also how activity is organized within the cerebral cortex. To this end, the attention axis was used to investigate the role of neuronal correlations between cerebral hemispheres during attention. One electrode array was implanted in each hemisphere in visual area V4, and for this analysis [6] a separate attention axis was constructed using neuronal responses from each of the two arrays. Trial-by-trial correlations in attention axis projections for the two cerebral hemispheres—using either the original or refined (Fig 6) attention axes—did not significantly deviate from zero between hemispheres (open bars, Fig 6). We also compared within-hemisphere correlations by randomly assigning the neurons recorded from each array into two groups and constructing an attention axis for each group. Unlike between-hemisphere changes, changes in position on the within-hemisphere attention axis were strongly correlated regardless of the behavioral outcome (filled bars, Fig 6). Between-hemisphere correlations were not detected when measured using either the original or revised attention axes, suggesting independent processing mechanisms for visual attention in each hemisphere [5].

## Discussion

We previously developed a novel method for measuring trial-by-trial changes in attentional state using activity recorded from small populations of neurons in animals performing a change detection task [5]. Conventional single-electrode neuronal measures of visual attention rely on activity averaged over many trials to obtain a reliable estimate of attentional state. The attention axis uses the responses of many neurons to obtain an estimate of attention on each trial. The ability to estimate attention at each moment makes it possible to study the dynamics and behavioral consequences of attention within brief periods.

However, we found that the original attention axis [5, 7] is susceptible to specific artifacts that can obscure results or suggest differences when none exist. At least two factors can bias the attention axis as it was originally defined. First, overlapping population responses between the two attention conditions can result in inversion of the attention axis polarity and, subsequently, sampling biased towards zero on missed trials (Fig 1). Second, a different bias on the attention axis occurs for multidimensional data and even for nonoverlapping distributions (Fig 2). In principle, these biases could be quantified and corrected if the properties of the relevant distributions are known, as in our reduced example in Fig 1. But such a correction is impossible for experimental data given the nature of neuronal responses.

These two biases can be avoided if the attention axis is constructed using responses that are independent from those that are analyzed. In our experiment, using Hit responses to stimuli presented two stimuli before the stimulus change (“n-2”) instead of the responses immediately preceding the stimulus change (“n-1”) achieved this goal. Responses to the n-1 stimulus on Hit and Miss trials can then be projected on the n-2 axis. Any biases act equally on all responses from n-2 trials, allowing uncompromised measures of differences in neuronal activity as a function of attentional state (Fig 3). Simulations suggest that this approach is reliable and unbiased until the difference between mean responses in the two conditions becomes very small compared to the noisiness of the population response (d' < 0.1).

Re-analysis of our previous work [5, 6] shows that our main conclusions remain valid (Figs 4–6). Position on the revised attention axis continues to show a strong correlation with behavioral performance (Fig 4). Neuronal modulation by attention is associated with changes in the slope and threshold of psychometric functions (Fig 5). Finally, positions on the attention axis constructed using responses across hemispheres remained uncorrelated even when using the revised attention axis (Fig 6). In all cases, the magnitudes of the reported effects are reduced using the revised attention axis. This is likely because some of the previously reported values arose from the artifactual biases described here. This is supported by the fact that the mean responses to attend-left and attend-right hits differed only by 8.6% in the neuronal data [5]. Such a difference would have resulted in a d' between the conditions that was only slightly greater than 0.2, a value where bias in the original attention axis would be expected to have some effect (Fig 3A). Nevertheless, the results with the revised attention axis could differ from those with the original attention axis even if the original measures were in fact unaffected by bias. Because the revised axis examines responses to one stimulus (n-1) using an attention axis constructed from responses to a different stimulus (n-2) and then relates the outcome to detection of a change in still another stimulus, it might be noisier than the original attention axis, which made use of neuronal and behavioral responses involving only two stimuli. A noisier measure would reduce the magnitudes of the reported effects in the way described here. Thus, we cannot be sure whether the original attention axis yielded larger effects because it included bias, or because it was a less noisy measure. Whatever the case, only the results from the revised attention axis can be considered reliable.

For decades, measures of population activity have played an important role in understanding limb movements and motor planning [8–10]. Multi-electrode recordings increase the amount of activity that can be monitored during a short interval and facilitate the analysis of the dynamics of the neural control of movement. The increasing use of multi-electrode recordings in visual cortex and associated regions will further test existing tools for interpreting population responses. The attention axis, as extended here, is one tool for monitoring visual attention or other arbitrary cognitive states. We hope that this approach and others like it [4, 11] will help reveal new perspectives on the interaction of individual neurons and behavior at relevant timescales.
